# Psychotropic drugs cross-reactivity with amphetamines in a FAERS sample: an international pharmacovigilance study

**DOI:** 10.1192/j.eurpsy.2022.875

**Published:** 2022-09-01

**Authors:** L. Giacovelli, V. Battini, M. Boscacci, C. Carnovale, B. Dell’Osso

**Affiliations:** University of Milan, Department Of Biomedical And Clinical Sciences “luigi Sacco”, Milan, Italy

**Keywords:** cross-reactivity, psychopharmacology, USDs

## Abstract

**Introduction:**

Urine Drug Screening (USD) is one of the most used techniques for drug testing. However, one of the main issues related to USD is the high frequency of cross-reactivity with other molecules. Amphetamines, because of their simple structures, are highly subjected to cross-reactivity with other molecules.

**Objectives:**

Our aim was to investigate and characterize the role of psychopharmacological drugs in the occurrence of false-positive amphetamine drug screening, by performing an international pharmacovigilance study through the *Food and Drug Administration Adverse Event Reporting System* (FAERS), in which user’s medication errors for drugs are reported in the form of Individual Case Safety Reports (ICSRs).

**Methods:**

All ICSRs recorded between 2010 and 2020 with a positive screening for amphetamine reported as adverse reaction in patients with a psychiatric diagnosis were included in the study. Duplicated records and ICSRs with missing values for age and gender, were excluded from the study.

**Results:**

Among 249 ICSRs involving false-positive amphetamine drug screening, 109 ICSRs reported psychiatric disorders and/or psychiatric drugs. In 83 (76%) cases, drugs were known for cross-react. 66 cases reported drugs known as “suspect”. 24% of cases reported unknown false-positive reactions: acetaminophen (5%), duloxetine (5%) and oxycodone (5%).

**Conclusions:**

The high cross-reactivity of psychotropic drugs with amphetamine testing in USDs may be linked to the neuromodulatory effect of these drugs, suggesting a similar molecular structure. In this perspective, antidepressants and amphetamines share a similar mechanism of action, maybe partially explaining why the most reported cross-reactions are with antidepressant (59%).

**Disclosure:**

No significant relationships.

